# Extracting chemical–protein interactions from literature using sentence structure analysis and feature engineering

**DOI:** 10.1093/database/bay138

**Published:** 2019-01-08

**Authors:** Pei-Yau Lung, Zhe He, Tingting Zhao, Disa Yu, Jinfeng Zhang

**Affiliations:** 1 Department of Statistics, Florida State University, Tallahassee, FL, USA; 2 School of Information, Florida State University, Tallahassee, FL, USA; 3 Department of Geography, Florida State University, Tallahassee, FL, USA

## Abstract

Information about the interactions between chemical compounds and proteins is indispensable for understanding the regulation of biological processes and the development of therapeutic drugs. Manually extracting such information from biomedical literature is very time and resource consuming. In this study, we propose a computational method to automatically extract chemical–protein interactions (CPIs) from a given text. Our method extracts CPI pairs and CPI triplets from sentences, where a CPI pair consists of a chemical compound and a protein name, and a CPI triplet consists of a CPI pair along with an interaction word describing their relationship. We extracted a diverse set of features from sentences that were used to build multiple machine learning models. Our models contain both simple features, which can be directly computed from sentences, and more sophisticated features derived using sentence structure analysis techniques. For example, one set of features was extracted based on the shortest paths between the CPI pairs or among the CPI triplets in the dependency graphs obtained from sentence parsing. We designed a three-stage approach to predict the multiple categories of CPIs. Our method performed the best among systems that use non-deep learning methods and outperformed several deep-learning-based systems in the track 5 of the BioCreative VI challenge. The features we designed in this study are informative and can be applied to other machine learning methods including deep learning.

## Introduction

Chemical–protein interactions (CPIs) play important roles in metabolism and the regulation of biological processes. The information contained in CPIs is essential toward understanding mechanisms of diseases and developing therapeutic drugs. Over the course of many years of biological and biomedical research, a significant amount of information on CPIs has been discovered and published in scientific literature. Since the CPI information published in scientific literature is stored as unstructured text that does not easily lend itself to automatic analysis and computation, a notable amount of scientific effort has been invested in manually extracting CPI information from literature and depositing it in a structured format into various databases. However, manually extracting CPI information is very time and resource consuming, and therefore relying on manual extraction methods is neither efficient nor sensible, given the speed of scientific publications. Consequently, developing computational methods that can automatically extract such information is of great interest and urgency.

A considerable number of computational methods have been developed to address a similar problem—extracting protein–protein interaction information (PPI) from literature (1–18). These approaches approached and addressed the problem using various techniques ranging from relatively simple co-occurrence, to rule-based pattern matching, to machine learning-based methods, which can be further enhanced by sophisticated natural language processing techniques. However, in contrast, few methods have been proposed to automatically extract the interactions or relations between genes/proteins and chemical compounds/drugs. A high-quality method to extract chemical–protein interactions will allow unstructured CPI information to be extracted efficiently from literature and deposited into databases to facilitate efficient querying of such information. The structured CPI information can also be used in integrative analysis of other biomedical data sets (19–24). Lastly, CPI extraction methods can be used in case studies of CPIs to get information on very specific types of chemical–protein interactions.

The BioCreative challenge VI has established a separate track titled ‘text mining chemical–protein interactions’ to promote research in this important area of biological test mining (25). In this track, the goal is to build a system to automatically extract the relations between chemical compounds/drugs and genes/proteins in PubMed abstracts, which involves (i) determining whether an interaction exists between a pair of chemical compounds and a proteins in a sentence, and if so, then furthermore (ii) identifying the type of interaction. The interaction types are detailed in ‘Section II Materials’. As a participant in this challenge, we developed a machine learning-based method for extracting CPI information from biomedical literature. By combining various sets of features from semantic patterns and sentence structures with several machine learning classifiers, our method is capable of identifying the CPIs from a given text with satisfactory accuracy. The results, which use data sets released by BioCreative VI, demonstrate that our CPI extraction method is effective.

The rest of the paper is organized as follows: we first describe the data used in this study, then present the proposed method and experimental results and end with a conclusion.

## Materials

The corpus used in this study comprises of PubMed articles. All chemical compounds and proteins entities are tagged by the BioCreative VI track 5 organizers. There are a total of 1632 abstracts, in which 41319 entities are tagged and 9995 CPIs are annotated in the training and development set. The test set contains 800 abstracts, 20828 tagged entities and 5744 CPIs. Each CPI is assigned to 1 of 10 chemical–protein interaction groups CPR (chemical-protein relationship):{1–10}, of which five groups are used for evaluation of models. These five groups carry useful information for downstream applications. They are up-regulator/activator (CPR:3), down-regulator/inhibitor (CPR:4), agonist (CPR:5), antagonist (CPR:6) and substrate (CPR:9). The task is to extract CPI as well as to identify the corresponding CPR type. References (25, 26) provide details on the definitions for each CPR and the guidelines used for CPI assignments in the corpus.

## Methods

Our CPI extraction method extracts potentially true CPI pairs and CPI triplets from a given text. A CPI pair is defined as a chemical entity and a protein entity in the same sentence, and a CPI triplet is defined as the combination of a CPI pair with an interaction word in the same sentence. Our CPI extraction method first builds models for CPI pairs and CPI triplets separately, then combines them to form a final model. The process begins by constructing all possible CPI pairs and CPI triplets from entities mentions in a sentence. For each pair or triplet, feature sets were extracted from both the semantic pattern and the dependency graph of the sentence. The semantic pattern contains useful information on how the interaction between chemicals and proteins is described in the sentences. The dependency graph provides information on how words are interconnected in the sentence. These features are used in machine learning classifiers to predict the corresponding CPR type of a CPI pair or a CPI triplet.

Several classifiers were first trained on training set, then evaluated on the development set. Classifiers with top F_1_ scores were selected for model building. The selected classifiers were retrained on the combined training and development sets before making predictions using the test set.

### Chemical–protein pairs and triplets construction

We used the chemical compounds and protein entities tagged by the BioCreative VI to construct CPI pairs and CPI triplets. A CPI pair is constructed with one chemical entity and one protein entity in a sentence. It is then labeled as 1 of 10 CPR types according to the relation annotations provided by the BioCreative VI. If a CPI pair is constructed but not included in the relation annotations, it will be labeled as CPR:10, which is defined as no interaction.

A CPI triplet is constructed as the combination of a CPI pair with one interaction word in the same sentence. The CPI triplet is then labeled as the CPR type of such CPI pair. If the sentence contains multiple interaction words, the same number of CPI triplets will be constructed, and they all share the same label. The interaction words in a sentence are informative for interaction extraction, since they may be used to describe the relationship between the chemical compound and the protein. We have manually built an interaction words dictionary based on our previous study (10). The dictionary contains 1155 interaction words. These interaction words were further manually mapped to the corresponding CPR type.

We constructed 18229 CPI pairs and 51460 CPI triplets from the training set, 11397 CPI pairs and 32150 CPI triplets from the development set, 15887 CPI pairs and 46403 CPI triplets from the test set. Table 1 reports the number of pairs and triplets in each type of CPR used for evaluation.

**Table 1:** Number of cases in each true CPR group

**Train set**	**CPR:3**	**CPR:4**	**CPR:5**	**CPR:6**	**CPR:9**
# pairs	761	2251	173	235	727
# triplets	2492	6452	486	732	1536
**Development set**	**CPR:3**	**CPR:4**	**CPR:5**	**CPR:6**	**CPR:9**
# pairs	548	1093	115	199	457
# triplets	1650	2936	400	673	881
**Test set**	**CPR:3**	**CPR:4**	**CPR:5**	**CPR:6**	**CPR:9**
# pairs	664	1658	185	292	644
# triplets	2120	4780	570	870	1371

### Features for model building

#### Semantic pattern

We extracted features based on our previous experiences and through reading some sentences in the training and development data sets. For example, we noticed that some interaction words in true CPI triplets are frequently located between two protein/gene entities of the triplets, and so we created a feature corresponding to the sequence of the three words in CPI triplets. As another example, we noticed that when a CPI pair is assigned to CPR:9, some related words—e.g. pathway, production, generate, synthesis—are frequently mentioned in the three words before or after the mention of the chemical compound or protein/gene. A feature was also created to catch such patterns. A detailed description of the features is provided in Table 2.

**Table 2:** Features of semantic pattern from a sentence

Features	Feature values	Comment
sp_type	Interger 0 to 7	Type 0: e1–iw–e2 (entity1–interaction word–entity2) Type 1: iw–e1–e2 Type 2: e1–e2–iw Type 3: the star shape with no other paths Type 4: the triangle shape Type 5: the star shape with a path between e1 and e2 Type 6: the star shape with a path between iw and e2 Type 7: the star shape with a path between iw and p1
SenLen	*Integer*	Number of words in a sentence.
steps_sp1	*Integer*	Number of edges in the shortest path between e1 and iw.
steps_sp2	*Integer*	Number of edges in the shortest path between iw and e2.
steps_sp3	*Integer*	Number of edges in the shortest path between e1 and e2.
pos_e1	*Integer*	Number of words which lie before e1.
pos_e2	*Integer*	Number of words which lie before e2.
pos_iw	*Integer*	Number of words which lie before interaction word.
NEntities	*integer*	Number of entities other than e1 and e2.
Significant	*y(presence), n(absence)*	Presence of *significant* within three words before e1 and three words after e2.
isBracket	*y(yes), n(no)*	Whether e1 or e2 is in (any kind of) brackets.
isSubstrate	*y(presence), n(absence)*	Presence of *product, pathway, generate, synthetic* within three words before e1 and three words after e2.
isAdjacent	*y(yes), n(no)*	Whether e1 and e2 are adjacent.

We also employed features proposed in an earlier study (10). These features capture certain grammar or language rules that people use to describe PPIs; we found that these rules are helpful in CPI extraction as well. For example, we included a binary feature that indicates the presence of negative words such as ‘not’, ‘incapable’ and ‘unable’ in the region covered by the CPI pair or triplet. This feature captures the negative meaning invoked by the sentence. Likewise, we included a binary feature that indicates the presence of conjunctions such as ‘although’, ‘therefore’, ‘whereas’, etc., in the region covered by the CPI pair or triplet. This feature captures compound meanings in the sentence.

#### Dependency graph

**Figure 1:**
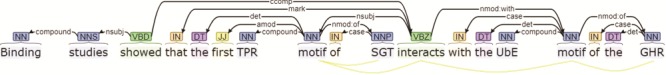
Grammatical dependencies graph.

The Stanford Neural Network Dependency Parser (27) was used to analyze sentence structure. The parser provides grammatical relationships among words in sentences, which are represented as dependency graphs where typed dependencies serve as edges between pairs of words (nodes in the graphs). Dependency graphs can be effectively used to extract entity relations. One direct way to extract the relation between two words in a sentence is to find the shortest dependency path (SDP) in the graph constructed for the sentence (28). It is reasonable to assume that SDP often contains necessary and sufficient information for identifying the relations of chemical and protein/gene entities mentioned in a sentence. Figure 1 shows an example of a dependency graph for the sentence ‘Binding studies showed that the first TPR motif of SGT interacts with the UbE motif of the GHR.’ The SDP between the two protein names, SGT and GHR, are colored as yellow. The arrows with texts, such as ‘compound’ and ‘nsubj’, are typed dependencies.

For better parsing accuracy, entities of a CPI pair were replaced with chemical (CHEM) and protein (PROT), respectively, in order to avoid the unnecessary complexity caused by multi-word terms when parsing a sentence. Other entities mentioned in the sentence were replaced with special symbols ‘CPT + interactor term identifier’. Table 3 shows an example of a sentence after the entity names have been replaced.

**Table 3:** Entities names replacement

**Sentence**	**PMID**	**Arg1**	**Arg2**
	**14507899**	**T15**	**T16**
Before	**P2Y(2) receptor** agonist **INS37217** enhances functional recovery after detachment caused by sub**retinal** injection in normal and rds mice.		
After	**PROT** agonist **CHEM** enhances functional recovery after detachment caused by sub**CPT10** injection in normal and rds mice.		

For each CPI pair, we obtained the SDP between CHEM and PROT. For each CPI triplet, two additional SDPs were obtained: CHEM to interaction word and interaction word to PROT. We next extracted features from the SDP, for example, the distance (number of words) between CHEM to PROT in SDP. This distance tends to have small values when there is a true interaction between a pair of CHEM and PROT. In addition, typed dependencies in SDP are helpful for distinguishing true CPI from false ones. For example, when SDP between CHEM and PROT has small distance value and contains ‘appos*’* (appositive) dependency, CHEM and PROT tend to be apposition, indicating that they have no interaction. We added one-hot encoded-typed dependencies as features to indicate which dependencies are included in the SPD.

### Three-stage model building

**Figure 2:**
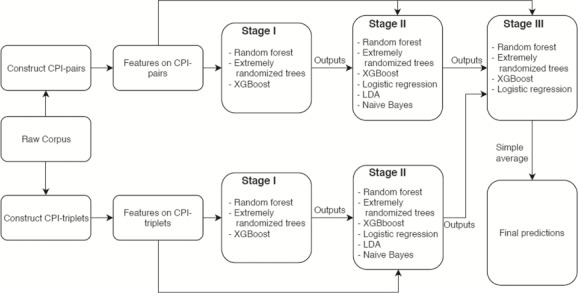
Flowchart of three-stage CPI extraction model.

We implemented a three-stage model building approach based on stacked generalization. Stacked generalization has proved to be effective in boosting the performance by reducing the generalization error (29, 30). At each stage, training data were first split into 10 disjoint folds. By taking one fold as holdout set and training a base model on the remaining folds, we obtained predictions for the holdout set and 10 different models. The holdout predictions will be used as an additional feature for the training data, and as a result, each training sample will have one more feature in the next stage of training. When predicting test data, we repeat the same procedure. Since there are 10 different models from each fold in the 10-fold training, each model will be used to predict test data and the predictions were averaged to give the additional feature for the test data, which is then used in the next stage of prediction. The complete flowchart of the three-stage model is given in Figure 2.

**Table 4:** Example for choosing CPI triplet

**Sentence**	PROT agonist CHEM enhances functional recovery after detachment caused by subCPT10 injection in normal and rds mice.		
**CPI-Triplet**	***Prediction***	***Score***	***Choose?***
CHEM-PROT-agonist	CPR:5	0.7234	Yes
CHEM-PROT-enhances	CPR:3	0.5118	No
CHEM-PROT-caused	CPR:9	0.3841	No

**Table 5:** F_1_ score of models at stage II

**Models**	***Include Stage I***	***Development set***	***Test set***
Triplets	Yes	0.5615	0.5558
	No	0.5284	0.5225
Pairs	Yes	0.5533	0.5275
	No	0.5192	0.4981

**Table 6:** Best F_1_ score of each team

**Team rank**	**Deep learning based**	**F**_**1**_ **score**	**Precision**	**Recall**
1 [39]	Y	0.6410	0.7266	0.5735
2 [40]	Y	0.6141	0.5610	0.6784
3 [41]	Y	0.6099	0.6608	0.5662
4 [42]	Y	0.5853	0.6704	0.5194
Our system	N	0.5671	0.6352	0.5121
6 [43]	Y	0.5181	0.5738	0.4722
7 [44]	Y	0.4948	0.5301	0.4639
8 [45]	Y	0.4582	0.4718	0.4453
9 [46]	Y	0.3839	0.2696	0.6663
10 [47]	N	0.3700	0.3387	0.4078
11 [48]	N	0.3092	0.2932	0.3271
12		0.2195	0.1618	0.3409
13 [49]	Y	0.1864	0.6057	0.1102

Tuning hyper-parameters and feature selection were performed at each stage. The hyper-parameters were tuned using randomized search. Under randomized search, the computational cost can be independent of the number, as well as the possible values, of the hyper-parameters (31). We performed randomized search for 10 different hyper-parameter settings with 3-fold cross validation, where each setting is sampled from a distribution over the possible hyper-parameter values. Recursive feature elimination (32) with 3-fold cross validation was implemented to select an optimal number of features.

**Table 7:** Confusion matrix and performance by CPR

	**Prediction**						
		**CPR:3**	**CPR:4**	**CPR:5**	**CPR:6**	**CPR:9**	**Other CPR**
**True**	**CPR:3**	305	59	1	0	4	296
	**CPR:4**	41	1085	2	1	6	526
	**CPR:5**	2	1	87	3	1	101
	**CPR:6**	0	4	2	172	0	115
	**CPR:9**	15	31	0	0	122	476
	**Other CPR**	198	426	22	27	72	-
	**F1 score**	0.498	0.665	0.5649	0.6964	0.2874	-
	**Precision**	0.5446	0.6773	0.7699	0.8557	0.5951	-
	**Recall**	0.4586	0.6532	0.4462	0.587	0.1894	-

#### Stage I

At stage I, three base models were trained on CPI pairs and CPI triplets in parallel to predict whether a sample belongs to one of the CPR types (CPR:{3, 4, 5, 6, 9}). The three base models for binary classification were the following: Random Forest (33), Extremely Randomized Trees (34) and XGBoost (35). The output rank-normalized scores (predicted probabilities) were stored for use at stage II.

#### Stage II

Base models for multiclass classification were trained on CPI pairs and CPI triplets in parallel to predict which CPR type a sample belongs to. The following three additional base models were used: Logistic Regression, Linear Discriminant Analysis and Naive Bayes (36). The scores for each CPR type, and the CPR type with highest score, from each base model were stored for use in the next stage. For those CPI triplets from a CPI pair due to multiple interaction words, we chose the one which results in the highest score for such CPI pair. Table 4 shows an example of choosing a CPI triplet constructed from the CPI pair in Table 3.

#### Stage III

At the final stage, the original features on CPI pair and the outputs at stage II were combined. Logistic Regression, Random Forest, Extremely Randomized Trees and XGBoost were implemented. The scores of each CPR type output from four classifiers were averaged, and the maximum was taken to be the final prediction.

## Results

Table 5 reports the F_1_ score of the models at stage II. It shows that scores from stage I are helpful for obtaining more accurate predictions of CPR type. In development set, the F_1_ scores increase by over 3% when the scores from stage I are included in the models. This improvement is also found in the test set when including the scores from stage I. In addition, introducing interaction words into CPI pairs to form CPI triplets is also helpful. Models built on CPI triplets achieve higher F_1_ scores than those built on pairs.

Table 6 shows the best performance of each team, as a team may have multiple submissions. The recall values are relatively low for all teams with a median value of 0.4722. This indicates that the CPI extraction remains very challenging. Our method obtained over 0.63 in precision and over 0.51 in recall, resulting in an F_1_ score of 0.56. Our system performed the best among those using non-deep learning methods. In addition, our system outperformed five systems that are based on deep learning. This shows that our models are effective, and the hand-crafted features used in the models are useful for CPI extraction.

Table 7 shows the confusion matrix and the performance divided by each CPR type. The diagonal indicates the number of correctly extracted interactions. CPR:5 (agonist) and CPR:6 (antagonist) have less confusion with other interactions. CPR:3 (up-regulator) and CPR:4 (down-regulator) are mainly confused with each other. It is relatively difficult to extract CPR:9 (substrate). Many annotated CPI assigned to CPR:9 cannot be identified by the model, resulting in the lowest F_1_ score and recall.

## Conclusion

In this study, we proposed a method to extract CPIs mentioned in biomedical literature. We constructed CPI pairs and CPI triplets, designed various sets of features from semantic pattern and sentence structure analysis and implemented a three-stage approach combining several machine learning classifiers. When tested on BioCreative VI track 5 data, our three-stage model achieved satisfactory performance. The source code is available at https://github.com/PeiYau-Lung/ChemProtBioCreativeVI.

There are several directions we can take to further improve our method. First, since our method heavily relies on hand-crafted features, we can improve it by extracting features that model more semantic patterns shared by various corpora. This can be done by reading sentences that contain the misclassified CPI. Second, all the CPI triplets formed by a CPI pair with different interaction words in a sentence shared the same label of such CPI pair, where some of the triplets were certainly mislabeled. This can be addressed in the future by selecting one and only one of them to assign the right label, assuming that only one triplet describes the interaction. This can be addressed by building another model to decide which triplet to be used for CPI extraction. This situation can also be addressed with multiple instance learning, which puts all the triplets of a pair into a bag and assume that at least one case in the bag is true (34, 37, 38).
